# Attitudes towards mandatory vaccinations during the COVID-19 pandemic in Bulgaria

**DOI:** 10.1093/eurpub/ckac130.229

**Published:** 2022-10-25

**Authors:** S Hadzhieva, R Chamova, E Ivanova, N Radeva, M Rohova, NL Mihaylov, Ts Paunov, M Kolarova, R Pancheva

**Affiliations:** 1 Department of Hygiene and Epidemiology, Faculty of Public Health, Medical University, Varna, Bulgaria; 2 Department of Disaster Medicine and Maritime Medicine, Faculty of Public Health, Medical University, Varna, Bulgaria; 3 Department of Economics and Health Care Management, Faculty of Public Health, Medical University, Varna, Bulgaria

## Abstract

**Background:**

Vaccinations are an essential public health strategy to control preventable diseases. A much-discussed approach to increase coverage is mandatory vaccination; however, its legitimacy and effectiveness were put to question during the COVID-19 pandemic. As of March 1,2022, Bulgaria had one of Europe's lowest immunization coverage rates against COVID-19. Only 29.3% of Bulgarians had completed COVID-19 vaccination, compared to 71% in the EU and EEA, and the country ranked last in number of booster doses (9.9% vs 51.4 %). This study aims to investigate the public's attitudes toward proposed mandatory COVID-19 vaccination and toward the long-standing mandatory child immunization schedule in the context of the COVID-19 pandemic.

**Methods:**

An online cross-sectional survey was conducted in April 2022 using a self-administered anonymous questionnaire to collect information on sociodemographic characteristics, vaccination status and attitudes toward mandatory vaccination to COVID-19 and the mandatory childhood immunization schedule.

**Results:**

Out of 1433 reached respondents, 1200(84%) completed the survey. The largest relative share of respondents is between 35-44y-33.3%; 72.7% were women; mainly highly educated (50.8%), and vaccinated participants (59.3%). There is a significant and large difference between vaccinated and unvaccinated regarding the full support of mandatory COVID vaccinations (46.1% vs 1.8%), and regarding mandatory child vaccinations (77.9% vs 50.4%). Mandatory childhood vaccination schedules are supported by 88.7% of those in favor of obligatory COVID-19 vaccinations and 56% of those who oppose them (p < 0.001). Significance is preserved after adjustment for vaccination status.

**Conclusions:**

Public health authorities need to develop well-organized vaccination campaigns in which accurate evidence-based information is adequately disseminated with respect to individuals’ autonomy. More research on the determinants of vaccination attitudes in Bulgaria is necessary.

**Key messages:**

- Mandatory vaccination programs should be proceeded by investigation of the social readiness for acceptance.
- Exploration of cultural specificities to influence vaccination decision is needed.

